# Autophagy Quantification and STAT3 Expression in a Human Skin Organ Culture Model for Innate Immunity to Herpes Zoster

**DOI:** 10.3389/fmicb.2018.02935

**Published:** 2018-12-05

**Authors:** Erin M. Buckingham, James Girsch, Wallen Jackson, Jeffrey I. Cohen, Charles Grose

**Affiliations:** ^1^Virology Laboratory, Children’s Hospital, University of Iowa, Iowa City, IA, United States; ^2^Laboratory of Infectious Diseases, National Institute of Allergy and Infectious Diseases, National Institutes of Health, Bethesda, MD, United States

**Keywords:** varicella-zoster virus, pseudorabies virus, herpes simplex virus, interleukin-6, ATG5, autophagosome, HSV ICP34.5, Imaris software

## Abstract

The goal of this project was to document the autophagy response in human neonatal skin organ culture (SOC) after infection with varicella-zoster virus (VZV). The VZV-infected SOC model has attributes of herpes zoster, in that an injection of virus into the skin is analogous to exit of virus from the sensory nerve termini into skin during herpes zoster. Cultures were maintained for 28 days and periodically examined for an autophagy response by quantitation of autophagosomes with Imaris software. Expression of the STAT3 protein was plentiful in the VZV-infected SOC. Abundant autophagy was observed in VZV-infected SOC between 14 and 28 days after infection, while autophagy in mock-infected SOC was minimal (*p* = 0.0003). The autophagic response after infection of SOC with a recombinant VZV genome containing the herpes simplex virus ICP34.5 neurovirulence gene was similar to wild-type VZV (*p* = 0.3). These results suggested that the VZV-infected SOC system resembled biopsy data from herpes zoster infection of skin. An enhanced autophagy response has now been reported after infection with two additional alpha herpesviruses besides VZV, namely, pseudorabies virus and duck enteritis herpes virus; both lack the ICP34.5 protein.

## Introduction

Herpesviruses are ancient viruses that have retained autophagy as a modulator of innate immune mechanisms (Grose, 2012; Moreau et al., 2015). varicella-zoster virus (VZV) is one of nine human herpesviruses (Davison, 2010).VZV is a human pathogen that causes the primary infection varicella in children and reactivates later in life as zoster or shingles (Weller, 1983). Abundant autophagy has now been detected after infection with VZV and two other alpha herpesviruses: pseudorabies virus (PRV) and duck enteritis herpes virus (Grose et al., 2016; Yin H.C. et al., 2017; Xu et al., 2018).

In earlier studies, we have demonstrated that VZV infection induces both an unfolded protein response (UPR) and an autophagy response (Carpenter et al., 2011; Carpenter and Grose, 2014). We have observed that VZV-induced autophagic flux is easily detectable in infected monolayers, without evidence of a block (Buckingham et al., 2014, 2015). In this manuscript, we describe our adaptation of the human skin organ culture (SOC) model to further assess the innate immune response to VZV infection (Taylor and Moffat, 2005; Jarosinski et al., 2018). We proposed that VZV infection of human SOC would provide a more accurate representation of innate immunity than analysis solely of infected monolayer cultures. Many earlier VZV studies were carried out after infection of human skin xenografts inserted under the skin of the severe combined immunodeficient mouse (Moffat et al., 1998; Arvin, 2006). The human SOC model is a simpler methodology for VZV infection of human skin that obviates the need for a severe combined immunodeficient mouse (Jarosinski et al., 2018). The chronology of the VZV infectious cycle is very similar in both skin models.

Because of previously documented differences in VZV pathogenesis between cultured cells and skin xenografts in the mouse model, we first examined transcription of antiviral innate immune transcripts in mock-infected SOC and infected SOC. We found unexpectedly that both interleukin-6 (IL-6) transcription as well as protein expression were upregulated following VZV infection (Jarosinski et al., 2018). Recent evidence points to IL-6 as a keystone immunomodulatory cytokine in both healthy and diseased tissues, including its role as a pro-autophagy cytokine in the phosphorylated signal transducer and activator of transcription 3 (STAT3) pathway (Xue et al., 2016). Since elevated IL-6 transcription was not observed in VZV-infected fibroblast monolayers, this discovery would not have been made without the studies in the SOC model. In this report, we further assess and quantify the autophagy response in SOC after infection with wild type VZV and recombinant VZV containing the herpes simplex virus 1 (HSV1) ICP34.5 neurovirulence gene.

## Materials and Methods

### Viruses and Cells

VZV-32 is a low passage laboratory strain; its genome has been completely sequenced (GenBank DQ479961.1) (Peters et al., 2006). rVZV/34.5 is a recombinant virus, in which herpes simplex virus 1 (HSV1) ICP34.5 gene (F strain) previously cloned into a pSG5 expression vector (Stratagene) was cut out along with its SV40 early promoter and poly A signal and inserted into rVZV-Oka (Cohen and Siedel, 1993). MRC-5 human fibroblast cells were grown on coverslips in six well tissue culture plates in Minimum Essential Medium (MEM; Gibco, Life Technologies) supplemented with 7% fetal bovine serum (FBS), L-glutamine, non-essential amino acids, and penicillin/streptomycin. When monolayers were nearly confluent, they were inoculated with trypsin-dispersed VZV-infected cells at a ratio of one infected cell to eight uninfected cells (Grose and Brunel, 1978).

### Culture of Explant Skin Samples

De-identified foreskins from circumcision procedures were collected at the University of Iowa Hospital under an exempt Institutional Review Board protocol. As described (Taylor and Moffat, 2005; Jarosinski et al., 2018), we prepared the foreskin for culture by sterilizing the outside of the skin with 1 min incubation in 10% providone-iodine solution followed by rinsing in 70% ethanol and MEM. The skin was then divided into 6 mm round pieces with a biopsy punch and placed onto the mesh bottom of tissue culture inserts in a 12 well plate (Corning) containing 1.4 ml of complete MEM supplemented with nystatin and ciprofloxin. Thereafter, the plate was incubated at 32^o^C in a humidified incubator with 5% CO_2_. We have carried out 9 independent explant procedures.

### Infection and Sectioning of Explant Skin Samples

After 24–48 h in culture, pieces of explanted human skin were inoculated with cell associated VZV. A VZV infected monolayer (25 sq. cm.) at 70–80% cytopathology was trypsin-dispersed, sedimented by low speed centrifugation and resuspended into 1 ml of MEM. Each skin piece was pierced to the depth of 1 mm in multiple sites with a hypodermic needle and then an aliquot (500,000 cells in 200 μl) of VZV-infected cells was injected onto the surface of the epidermis and allowed to penetrate into the holes created by piercing the skin surface. Infected skin explants were then incubated for as long as 28 days. Mock infections were handled similarly except for inoculation with uninfected cells. Preparation of the skin samples for cryosectioning and subsequent immunohistochemistry (IHC) protocols have been described in detail (Jarosinski et al., 2018).

### Imaging and Quantitative Analysis of Autophagy by Confocal Microscopy

Slides containing the skin samples were prepared for confocal microscopy by published methods (Carpenter et al., 2008). The primary antibody (1:1000) was added for 2 h at ambient temperature and overnight at 4^o^C; the secondary antibody (1:1250) and Hoechst 33342 DNA stain (1:1000) were added for 2 h. All samples were viewed on a Zeiss 710 confocal fluorescent microscope. Murine monoclonal antibody (MAb) 3B3 against VZV gE, MAb 233 against gC and MAb 251D9 against the capsid protein were produced in this laboratory (Grose et al., 1983; Friedrichs and Grose, 1986; Storlie et al., 2008); rabbit antibody against microtubule-associated protein light chain 3 (LC3) was obtained from Santa Cruz (sc-28266) and mouse MAb against STAT3 was obtained from ThermoFisher Scientific (13-7000). Rabbit monospecific antibody against HSV1 ICP34.5 was given to us by Dr. Ian Mohr (New York University).

Quantitation of autophagosomes by visual inspection of 2D confocal micrographs is highly specific when combined with appropriate controls (Klionsky et al., 2007; Carpenter et al., 2011). Quantitation has been further improved by the Imaris software program (BitPlane), which converts z-stacks of confocal images into 3D animations; the Spot function within Imaris software locates and enumerates autophagosomes within each 3D animation based on size and intensity threshold (Jackson et al., 2013; Lamb et al., 2017). We examined at least 3 separate images from each experimental group in order to determine the number of autophagosomes per cell in each image. The standard error of the mean (SEM) across each set of images was used to generate error bars. The *P* values were determined by unpaired, two-tailed Student’s *t* tests, using GraphPad Prism software.

## Results

### Expression of the True Late gC Protein in VZV Infected SOC

**FIGURE 1:**
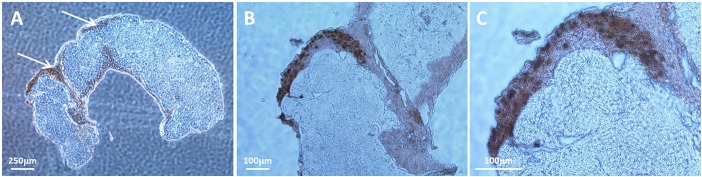
Visualization of a representative VZV-infection within epidermis of human skin explant. Pieces of human skin obtained with a punch that measured 6 mm in diameter were placed in culture medium. As described in Methods, each piece of skin was pierced multiple times with a syringe needle before the infected cell inoculum was layered onto the surface of the same explant. At 14 dpi, infection was analyzed by IHC after labeling with an antibody to a VZV small capsid protein. Since the capsid protein is found mainly in the nuclei of infected cells, the IHC pattern outlined the nuclei within an infectious focus in the SOC epidermis. Two piercing sites in the biopsy are marked with arrows **(A)**. Enlargements of panel **A** are shown in panels **B,C**. The infectious focus contains around 30 nuclei (dark brown color).

A major difference between VZV infection of cultured cells and human skin biopsies during herpes zoster is that mature viral particles are produced in human skin in abundance whereas most viral particles produced in cell culture are aberrant (Tournier et al., 1957; Storlie et al., 2008; Carpenter et al., 2009). A representative example of a VZV-infected SOC is shown in Figure 1. In this figure, the cells were prepared for IHC and labeled with an anti-capsid antibody because the nuclei at 14 dpi are filled with capsids. Cell-to-cell virus spread within the epidermis is designated by arrows (Figure 1A); higher magnifications of the capsid-labeled infected cells within the SOC are shown in panels B and C.

**FIGURE 2:**
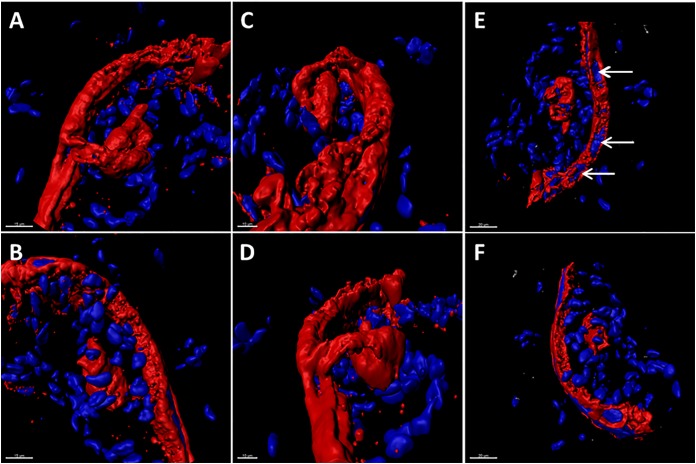
The true late phase of the VZV-infectious cycle in human skin explants. At 14 dpi, VZV infection was analyzed by confocal microscopy with subsequent creation of 3D animations of infected SOC by merging the z-stacks with Imaris software. In order to document that viral replication had entered the late phase of the VZV infectious cycle in human skin, the infectious foci in the skin were immunolabeled with antibody to the true late VZV gC glycoprotein (red color). Six different orientations of the infectious focus are shown in panels **A–F**. In panel **E**, VZV-induced syncytia are visible as clusters of nuclei surrounded by the gC glycoprotein (white arrows). Nuclei are colored blue by the Hoechst dye.

A marker of the late phase of the alpha herpesvirus infectious cycle is the true late protein gC (Honess and Roizman, 1974). Expression of VZV gC is sparse in cultured cells but plentiful in human skin during herpes zoster (Storlie et al., 2008). Therefore, we investigated the appearance of the gC protein in the VZV infected SOC by confocal microscopy. As seen in the 3D images of the infected SOC, gC expression was easily detected along the linear piercing sites in the skin explant created with the syringe needle (Figure 2). As demonstrated by 6 different orientations of the Imaris animation (panels A–F), VZV infection had spread cell-to cell via a piercing site into the epidermis by 14 dpi and then outward from this site. This outward spread into the epidermis was not uniform, rather the spread was meandering.

### Expression of STAT3 in the VZV Infected SOC

**FIGURE 3:**
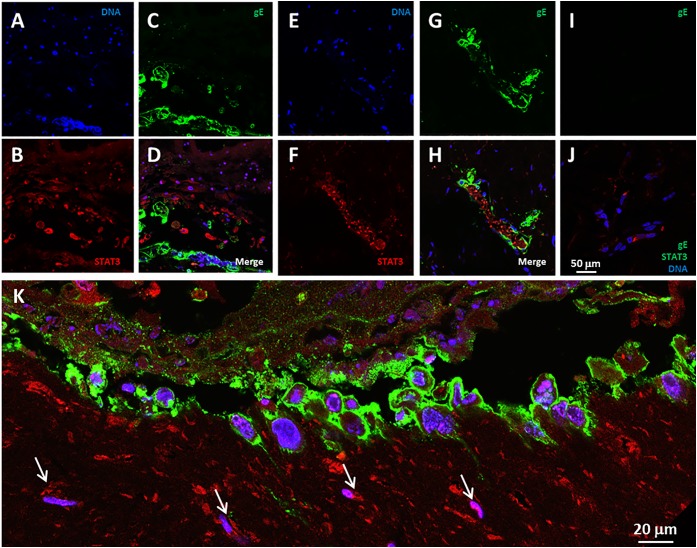
STAT3 expression in VZV infected human skin explants. **(A–H)** STAT3 expression at 14 dpi **(A–D)** and 28 dpi **(E–H)**. The panels show infectious foci outlined by VZV gE immunolabeling mainly in the outer cell membranes, with STAT3 positive cells in the vicinity of the infected cells. **(A,E)** Blue, Hoechst 33342. **(B,F)** Red, STAT3. **(C,G)** VZV gE, green. Panels **D,H** are the merged images of infected explants. **(I,J)** Control images of mock-infected skin explants. The white bar in panel**J** represents all prior panels. **(K)** A higher magnification landscape image of an infectious focus at 14 dpi with a merge of all colors. Arrows designate cells with immunolabeling for STAT3.

We previously detected elevated levels of the IL-6 protein in media overlying infected SOC (Jarosinski et al., 2018). A recent report has provided evidence that IL-6 can act as a pro-autophagy cytokine within the IL-6-STAT3 pathway (Xue et al., 2016). Therefore, we looked for evidence of STAT3 expression in the VZV infected SOC. We observed STAT3 immunoreactivity in the VZV-infected skin, in particular, STAT3 expression was found in bystander cells in the near vicinity of an infectious focus as well as within numerous cells of an infectious focus (Figures 3A–H). In uninfected skin, only a few randomly distributed STAT3-immunoreactive cells were seen, while no foci of reactivity were observed with the anti-VZV gE antibody (Figures 3I,J). The lower panel K represents a landscape image of another infectious focus at a higher magnification (Figure 3K).

### Quantitation of Autophagosomes in the VZV Infected SOC

**FIGURE 4:**
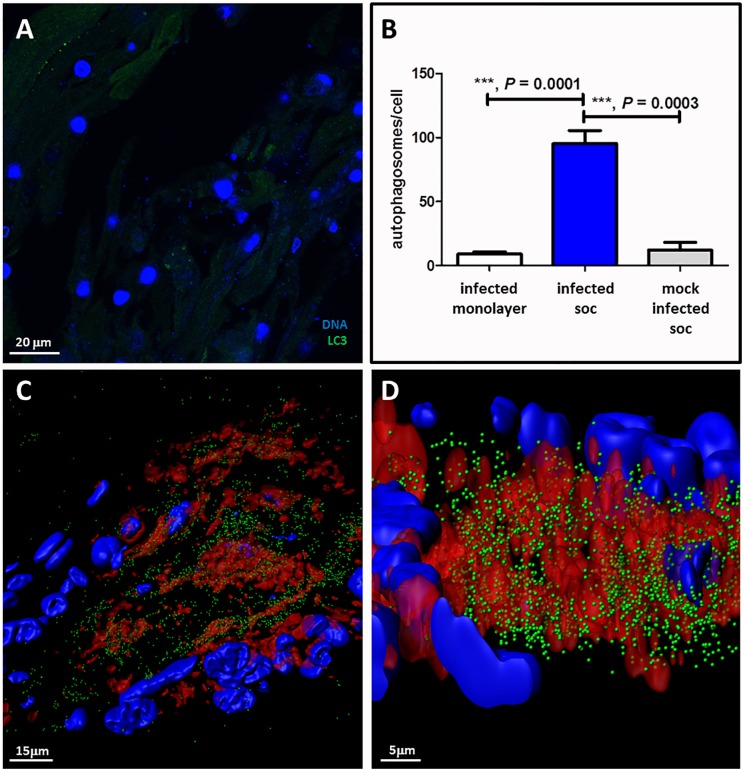
Autophagy in VZV-infected human skin explants. **(A)** Autophagosome formation in mock-infected skin explants. This micrograph is representative of micrographs taken during 6 different experiments. This micrograph includes 27 nuclei in the skin. **(B)** Graph of the number of autophagosomes per cell in infected monolayers versus infected human SOC versus mock-infected human SOC. Data in the infected SOC bar represent 3 separate experiments; data in the uninfected SOC bar represent 6 separate experiments. **(C)** Enumeration of autophagosomes after rendering z-stacks of 2D images of VZV-infected SOC into 3D images by Imaris software. Top-view frame from the 3D animation shows 3740 autophagosomes (green spheres) within the cytoplasm surrounding an infectious focus when the estimated diameter within the Imaris Spot function is set at 500 nm. **(D)** A more enlarged side-view of the prior animation seen in panel **C**. VZV gE, red; LC3, green; Hoechst stain, blue. Three asterisks ^∗∗∗^indicates highly significant result.

We also carried out a series of confocal microscopy experiments on the VZV infected SOC, in order to investigate the degree of autophagy (Figure 4). We first examined a mock-infected SOC during each of 6 experiments and observed a minimal number of autophagosomes (Figure 4A). After examination of numerous confocal images of VZV infected SOC, we concluded that we had visualized a very robust autophagy response. Measurement of changes in autophagy by enumeration of LC3-positive puncta is one of the most specific assays, especially when combined with analysis by the Imaris software program, which creates a 3D rendering of each z-stack of confocal micrographs as described in Methods. It was apparent that the maximal autophagy response in each cell within VZV-infected human skin was statistically greater than that seen in mock-infected skin (*p* = 0.0003) (Figure 4B). As expected, the number of autophagosomes counted within a 3D image of an infected cell was also greater than the number counted in a single 2D image (Figures 4C,D). Finally, we selected two images created by the Imaris animation. There were 3,740 autophagosomes detectable in the cytoplasm above the infectious focus when we set the estimated XY diameter of an autophagosome at 500 nm (Figure 4), much higher when performed on 3D renderings of cells rather than on 2D images seen in a confocal micrograph.

### Autophagy After SOC Infection With Recombinant VZV/34.5 Virus

**FIGURE 5:**
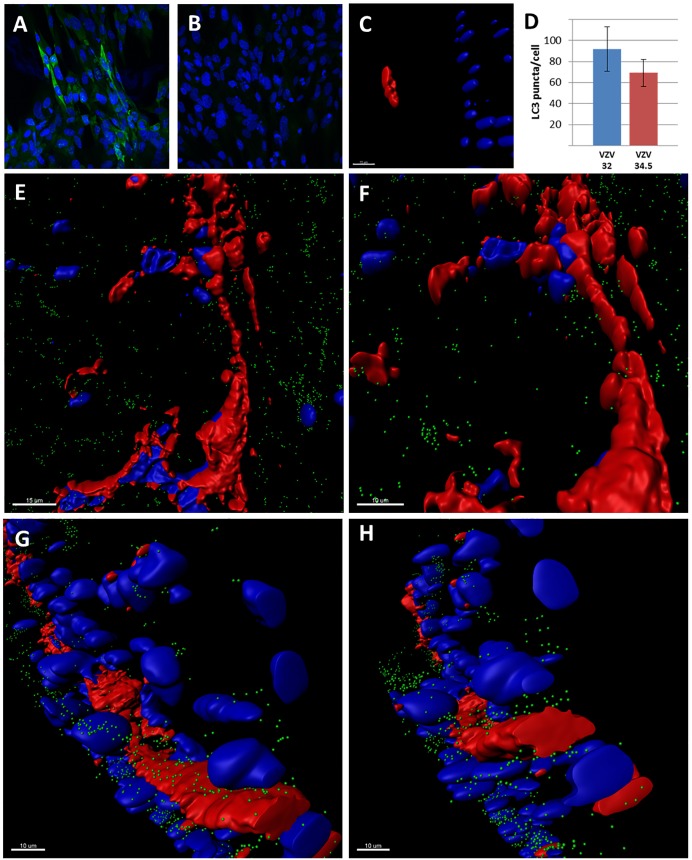
Studies with the recombinant VZV/34.5 virus. **(A)** Immunolabeling of the HSV1 ICP34.5 protein after infection with rVZV/34.5. The ICP34.5 protein is labeled green. **(B)** Control immunolabeling with the anti-ICP34.5 antibody in wild-type VZV infection. No ICP34.5 protein was detected. **(C)** Small focus of VZV/34.5 infection in SOC at 14 dpi. Larger infectious foci were first seen at 28 dpi. **(D)** Graph of autophagosome counts for VZV-32 and VZV/34.5 strains in SOC at 28 dpi. Six individual sections were examined. There was no statistical difference. **(E–H)** Imaris renderings of confocal micrographs in SOC infected with VZV/34.5 for 28 days. VZV-induced syncytia were apparent in each panel because VZV gE immunolabeling was detectable around multiple nuclei without evidence of any barriers by individual cellular membranes. VZV glycoprotein gE is labeled red; autophagic puncta are labeled green; nuclei are labeled blue.

After our analyses in SOC described previously as well as the above new data (Jarosinski et al., 2018), we carried out an experiment with a recombinant VZV genome containing the HSV ICP34.5 neurovirulence gene. In our earlier publication, we showed that infection of SOC with wild type VZV closely followed the SCID/human skin model: namely, VZV infection progressed and achieved near maximal distribution by 14 dpi. Although ICP34.5 contributes to HSV neurovirulence in the brain by attaching to Beclin-1 and suppressing autophagy (Orvedahl et al., 2007), the role of ICP34.5 in HSV1-infected non-neuronal tissues has been a subject of debate in the HSV1 literature (Yordy and Iwasaki, 2013). We propose that repeating similar experiments in a VZV/SOC system is an appropriate control in which to explore the effects of ICP34.5 on autophagy outside of the neuronal system. To this end, the ICP 34.5 gene was cloned into the varicella vaccine genome. We first documented that the ICP34.5 protein was expressed by immunolabeling the protein after rVZV/34.5 infection (Figure 5A). As in the SCID model of VZV infection, spread of varicella vaccine virus occurred more slowly in SOC than wild-type VZV (Moffat et al., 1998); at 14 dpi, there were only small foci of infection with the rVZV/34.5 virus (Figure 5C).

Thereafter, we examined the infected SOC culture at 28 dpi (Figures 5D–H). Time points beyond 21 dpi were not examined often in the SCID model. But at 28 dpi in SOC, we observed that the foci of infection with the rVZV/34.5 virus had enlarged and were similar in size to foci with wild type virus at 14 dpi (compare Figures 4, 5). Since wild type virus infection does not progress greatly between 14 and 28 dpi, the foci in a control experiment with wild type virus were similar in size to that of rVZV/34.5 at 28 dpi. Then, we obtained images from 6 individual infectious foci in order to quantitate puncta by confocal microscopy combined with Imaris software. Since the samples were labeled with antibody to VZV gE, the predominant VZV glycoprotein, we documented the extensive production of VZV gE throughout the cytoplasm of the infected SOC (Figures 5E–H). When we enumerated the puncta within individual infected cells by the Imaris Spot program, we found no significant difference in puncta per infected cell when wild-type and rVZV/34.5 infection were compared (Figure 5D; *p* = 0.3). We have previously shown that autophagy and LC3 production were not diminished in cells infected with vaccine virus (Buckingham et al., 2016). This further experiment documented that ICP34.5 when expressed within a VZV genome did not limit autophagosome production in human skin tissue.

## Discussion

A goal of the current study was to investigate autophagy in the human SOC model for herpes zoster. A prior investigation in the SOC model documented a dramatic increase in IL-6 transcription in VZV-infected skin explants (Jarosinski et al., 2018). IL-6 has multiple functions that involve both pro-inflammatory and anti-inflammatory effects (Hunter and Jones, 2015). Prior skin studies have shown an induction of IL-6 transcription in injured human keratinocytes (Sugawara et al., 2001). Our result also supports an earlier report that found IL-6 transcripts in skin biopsies from human subjects with herpes zoster (Uceyler et al., 2014). Increased levels of IL-6 also have been found in the cerebrospinal fluid of patients with vasculopathy in the brain (Jones et al., 2016), and IL-6 inhibits VZV replication in stem-cell derived neuronal cultures (Como et al., 2018). Further, IL-6 transcription is upregulated in the spleen during an avian herpesvirus infection, while increased IL-6 expression is found in the diseased eye after HSV1 infection (Heidari et al., 2010; Bryant-Hudson et al., 2014; Chucair-Elliott et al., 2016). The VZV IL-6 data are strongly supported by an independent investigation of cytokines present in the skin disease called “mad itch” found in animals infected with the closely related PRV, an alpha herpesvirus in the Varicellovirus genus (Laval et al., 2018). The PRV-induced systemic inflammatory response responsible for “mad itch” included elevated IL-6 protein levels in the mouse footpad.

The above VZV IL-6 data are relevant because a recent paper describes a previously unrecognized pathway between elevated IL-6 and autophagy. Hypoxia within a brain tumor stimulated IL-6, which acted upstream as an inducer of autophagy in glioblastoma tumor cells via the IL-6-STAT3-MIR155-3p-CREBRF-CREB3-ATG5 pathway (Xue et al., 2016). In addition to IL-6 studies, we have shown the importance of ATG5 in the VZV induced autophagy pathway (Buckingham et al., 2014). In a prior important study from another laboratory, VZV infection led to increased phosphorylation of STAT3 in skin xenografts; in turn, STAT3 activation was critical for VZV replication in the skin xenograft (Sen et al., 2012). We have confirmed STAT3 activation in infected SOC. Thus, we have now documented the presence of three of the six components of the above autophagy pathway.

Further, our autophagy quantification after infection of SOC with the rVZV/34.5 virus may support HSV1 data that the primary anti-autophagy effects of the ICP34.5 protein are recognized mainly in HSV1-infected neuronal tissues (Yordy and Iwasaki, 2013). A complementary hypothesis is that anti-autophagy effects of ICP34.5 may require or be facilitated by the presence of additional HSV1 proteins not expressed by the smaller VZV genome, for example, US11 (Lussignol et al., 2013). A more recent investigation suggests a non-autophagy mechanism to explain some proviral effects of ICP34.5, namely, that HSV1 ICP34.5 restricts the type 1 interferon response to HSV1 infection indirectly by sustaining expression of the immediate-early HSV1 immunomodulatory gene ICP0 (Manivanh et al., 2017). During VZV infection, the VZV ICP4 homolog (ORF62) is the dominant immediate-early protein, while the VZV ICP0 homolog (ORF61) plays an accessory role (Perera et al., 1992).

When these VZV data about IL-6/STAT3/ATG5 are considered together with the results from the glioblastoma/autophagy study, we conclude that the elevated levels of IL-6 may also facilitate elevated levels of autophagy in VZV-infected skin explants. In support of this hypothesis, we point out the robust autophagosome formation seen in the 3D images of our SOC model of VZV skin infection (Figure 4). There were ∼100 autophagosomes per cell in the syncytium with approximately 30 nuclei; this result matches closely with a prior imaging of a biopsy of a human zoster vesicle, where we also enumerated ∼100 autophagosomes per cell (Jackson et al., 2013). In earlier studies using 2D confocal micrographs, we had found a lower range of autophagosomes (10–20) per infected cell, while uninfected and unstressed cells had <4/ cell (Carpenter et al., 2011). The number of <4 puncta/cell is generally accepted in the autophagy literature as a standard marker for unstressed cells in 2D images (Klionsky et al., 2007).

Finally, we would like to point out papers that describe enhanced autophagy following infection with two other alpha herpes viruses: PRV and duck enteritis herpes virus (Yin H. et al., 2017; Yin H.C. et al., 2017; Xu et al., 2018). The data within these manuscripts were mutually supportive, for example, the similarities between innate immunity in response to VZV and PRV infection (Jarosinski et al., 2018; Laval et al., 2018). When autophagy was inhibited, viral titers decreased (Grose et al., 2015). With regard to VZV, we note that the abundant autophagy response observed in our SOC model of herpes zoster confirms the abundant autophagy previously reported in skin vesicle biopsies from patients with herpes zoster (Takahashi et al., 2009). We also point out that studies of innate immunity in cultured cells may not always reflect results in human tissue explants, for example, there is no Il-6 production in VZV-infected fibroblast cells while IL-6 production is detected after VZV infection of a human retinal pigment cell line (ARPE-19) (Graybill et al., 2017; Jarosinski et al., 2018). Similar experiments are needed in biopsies of PRV-infected swine and duck tissue infected with duck enteritis virus, in order to validate positive autophagy results in cultured cells.

## Author Contributions

CG provided overall supervision for the project. EB, JG, and WJ carried out experiments shown in Figures 1–4. JC provided the recombinant varicella virus used in Figure 5. All authors assembled the data, designed the figures, and contributed to writing the manuscript.

## Conflict of Interest Statement

The authors declare that the research was conducted in the absence of any commercial or financial relationships that could be construed as a potential conflict of interest.
